# Acute effects of empagliflozin on left atrial and ventricular filling parameters using echocardiography—a subanalysis of the EMPAG-HF trial

**DOI:** 10.1093/ehjcvp/pvaf003

**Published:** 2025-03-03

**Authors:** Jurgen Bogoviku, Tien Dung Nguyen, Julian Georg Westphal, Pawel Aftanski, Sven Moebius-Winkler, Franz Haertel, Sissy Grund, Ali Hamadanchi, Martin Busch, Paul Christian Schulze

**Affiliations:** Department of Internal Medicine I, Division of Cardiology, Angiology and Intensive Medical Care, University Hospital Jena, Friedrich-Schiller-University, Am Klinikum 1 Haus A, 07747 Jena, Germany; Department of Internal Medicine I, Division of Cardiology, Angiology and Intensive Medical Care, University Hospital Jena, Friedrich-Schiller-University, Am Klinikum 1 Haus A, 07747 Jena, Germany; Department of Internal Medicine I, Division of Cardiology, Angiology and Intensive Medical Care, University Hospital Jena, Friedrich-Schiller-University, Am Klinikum 1 Haus A, 07747 Jena, Germany; Department of Internal Medicine I, Division of Cardiology, Angiology and Intensive Medical Care, University Hospital Jena, Friedrich-Schiller-University, Am Klinikum 1 Haus A, 07747 Jena, Germany; Department of Internal Medicine I, Division of Cardiology, Angiology and Intensive Medical Care, University Hospital Jena, Friedrich-Schiller-University, Am Klinikum 1 Haus A, 07747 Jena, Germany; Department of Internal Medicine I, Division of Cardiology, Angiology and Intensive Medical Care, University Hospital Jena, Friedrich-Schiller-University, Am Klinikum 1 Haus A, 07747 Jena, Germany; Department of Internal Medicine I, Division of Cardiology, Angiology and Intensive Medical Care, University Hospital Jena, Friedrich-Schiller-University, Am Klinikum 1 Haus A, 07747 Jena, Germany; Department of Internal Medicine I, Division of Cardiology, Angiology and Intensive Medical Care, University Hospital Jena, Friedrich-Schiller-University, Am Klinikum 1 Haus A, 07747 Jena, Germany; Department of Internal Medicine III, Division of Nephrology, University Hospital Jena, Friedrich-Schiller-University, Am Klinikum 1 Haus A, 07747 Jena, Germany; Department of Internal Medicine I, Division of Cardiology, Angiology and Intensive Medical Care, University Hospital Jena, Friedrich-Schiller-University, Am Klinikum 1 Haus A, 07747 Jena, Germany

**Keywords:** Acute decompensated heart failure, Echocardiographic evaluation, Empagliflozin, SGLT-2 inhibition

## Abstract

**Background:**

Sodium-glucose co-transporter 2 (SGLT2) inhibitors improve prognosis in chronic heart failure as part of currently recommended therapeutic strategies. Transthoracic echocardiography (TTE) is frequently used to assess heart function and dimensions in acute heart failure to lead therapy and assess volume status. Immediate changes, especially of left heart haemodynamic parameters, measured by echocardiography in patients with acute heart failure treated with SGLT2 inhibitors, remain unknown.

**Aim:**

The aim of this pre-defined secondary analysis was to assess whether treatment with empagliflozin 25 mg/day in patients with acute heart failure improves echocardiographic parameters of load, left ventricular or right ventricular function.

**Methods and results:**

In the single-centre, prospective, double-blind, placebo-controlled EMPAG-HF trial, patients with acute decompensated heart failure (ADHF) were screened and randomized within 12 h following hospital admission to receive either empagliflozin or placebo in addition to standard medical treatment over 5 days. Sixty patients were enrolled and randomized irrespective of left ventricular ejection fraction or diabetes. All patients received 2D TTE on admission (tB = at baseline) and after completing the study treatment (tC = time after completing study medication) (according to study design). The recorded loops were analysed using dedicated software (Image-Arena™ Version 4.6; TomTec Imaging Systems). After 5 days of treatment, patients in the empagliflozin cohort showed a relevant decrease in left atrial volume [LAV: ∆tB-tC = 30.9 ± 27.4; 95% confidence interval (CI) 20.1–41.7) compared to placebo ∆tB-tC = 10.5 ± 26; 95% CI 0.4–20.5; *P* = <0.001] and left atrial end-systolic volume index (LAESVI: ∆tB-tC = 15.7 ± 15.1; 95% CI 9.8–21.6 vs. placebo ∆tB-tC = 9.7 ± 10.2; 95% CI 5.7–13.6; *P* = 0.016) compared to placebo.

**Conclusion:**

Immediate addition of empagliflozin to standard therapy improves echocardiographic parameters of LAV in patients following recompensation of ADHF.

## Graphical Abstract

**Graphical Abstract:**
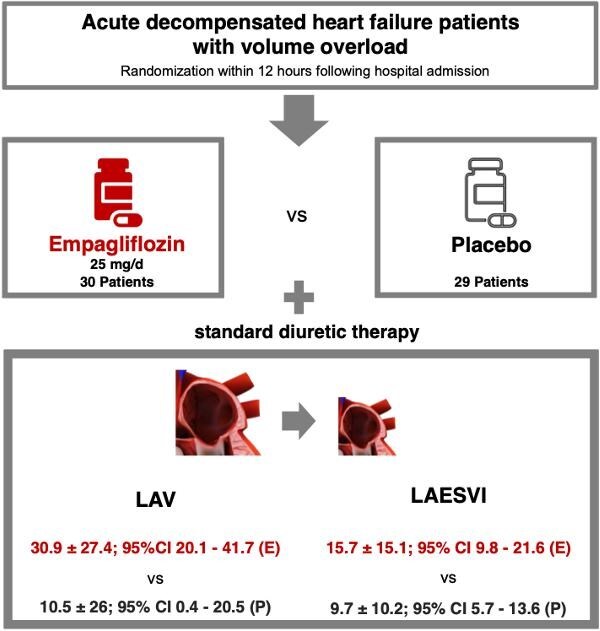
Central illustration. Main findings of the echocardiographic evaluation of early initiation of empagliflozin (25 mg/day) in ADHF (LA-Volume, left atrial volume; LAESVI, left atrial end-systolic volume index; E, empagliflozin; and P, Placebo).

## Introduction

Heart failure represents a leading cause of hospitalization with high morbidity and mortality worldwide.^1–4^ Acute decompensated heart failure (ADHF) is the most critical aspect of this complex syndrome.^5^ The inability of compensatory mechanisms to preserve a steady state resulting in clinical signs of congestion, volume retention, and increased filling pressures such as dyspnea, fatigue, chest pain, peripheral oedema, or distended jugular veins, is the most frequent cause for urgent and unplanned hospital admission in patients of >65 years of age.^6^ In the last decades, no new therapy has been implemented in the acute setting. Patient prognosis depends on the effectiveness of decongestive therapeutical pathways^2,6^ with focus on the reduction of left ventricular loading through depletion of preload.^7^ Loop diuretics represent the cornerstone of therapeutic regimen^8^ in acute heart failure characterized by diuretic response and resistance^9–11^ which predict therapeutic efficiency. Other strategies such as haemofiltration or the addition of inotropic agents in case of hypoperfusion increase diuresis and decongestion. Of note, the improvement of urine output without worsening renal function represents a pivotal challenge for novel therapies.

Sodium–glucose co-transporter 2 (SGLT2) inhibitors reduce the risk of cardiovascular death and hospitalization in patients with stable chronic heart failure.^12–15^ Their role in an acute setting as addition to diuretic therapy is still unknown. The EMPAG-HF (*EMPAG*liflozin in ADHF—NCT04049045 -empagliflozin vs. placebo—double-blind, placebo-controlled trial) study tested the effect of an early initiation of SGLT2 inhibitors in the enhancement of diuresis without the induction of acute renal injury. According to study design, transthoracic echocardiography (TTE) was performed on admission and after completing treatment. TTE represents a key non-invasive evaluation of ejection fraction, cavity dimensions, filling parameters, and valvular pathologies and can not only improve the diagnostic accuracy but also guide and monitor further therapeutic interventions.^16,17^

We performed a pre-defined echocardiographic subanalysis to assess changes in cardiac imaging parameters in a prospective, randomized, and placebo-controlled setting.

## Methods

**Figure 1:**
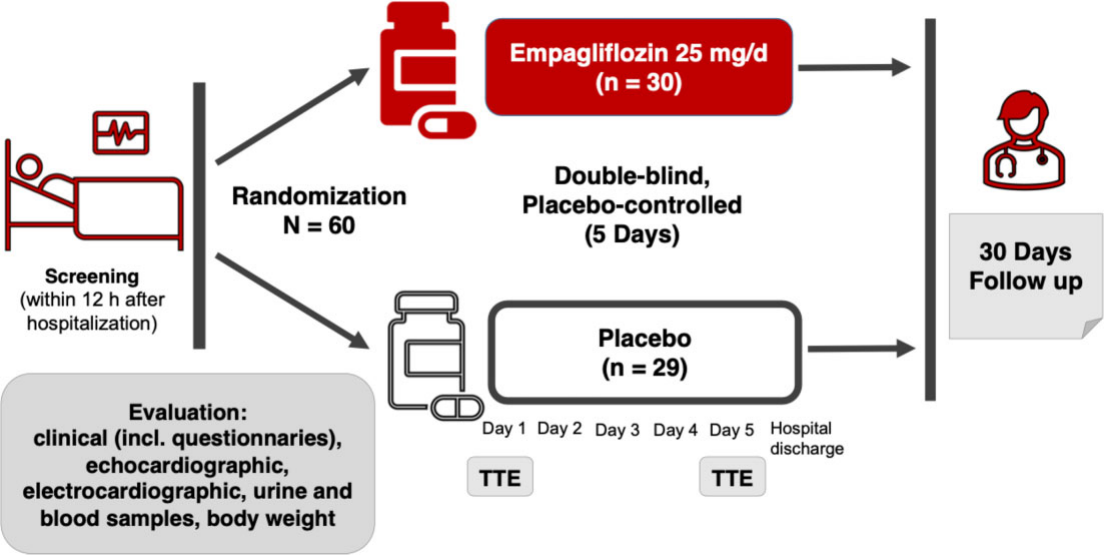
Study design of the EMPAG-HF trial (*EMPAG*liflozin in ADHF). One patient in the placebo cohort had to be excluded after randomization before administration of the first dose of trial medication because of acute ECG changes and need for urgent cardiac catheterization.

The EMPAG-HF (NCT04049045) trial was a prospective double-blind, placebo-controlled trial that tested the early and immediate effects of SGLT2 inhibitor empagliflozin compared to placebo in addition to standard medical care on urine output as well as acute renal injury. Twenty five milligrams (study medication vs. placebo) was daily administered within 12 h of hospital admission for 5 days in addition to standard medical care (*Figure 1*).

The local Ethics Committee of the University Hospital Jena approved the protocol. All patients gave written informed consent. Comprehensive TTE, focusing on left heart haemodynamic parameters, was performed on admission and after completing treatment (baseline; day 5). The main results of the EMPAG-HF trial have been previously published.^18^

### Patient cohort

Patients who were admitted to our institution between June 2019 and April 2020 because of ADHF were screened for eligibility within 12 h. Ultimately 60 patients between 18 and 85 years old with elevation of N-terminal pro-brain natriuretic peptide (NT-proBNP) over 300 pg/mL were enrolled. Furthermore, the inclusion of probands was irrespective of aspects such as the aetiology of heart failure, ejection fraction, the presence of diabetes mellitus type 2, or impaired glucose tolerance. Pregnancy for women of childbearing potential was evaluated through testing and adequate documentation of correct use of contraception. Exclusion criteria consisted of the presence of diabetes mellitus type 1, current SGLT2 inhibition, intolerance or contraindication to furosemide or empagliflozin, chronic kidney disease with estimated glomerular filtration rate (eGFR) <30 mL/min/1.73 m^2^ or acute kidney injury stage 2, acute heart failure without signs of congestion, indication for urgent angiography or administration of iodine based contrast agent within the next 6 days, instable patients (inotropy/vasopressor or mechanical support dependant), planned operations, alcohol abuse (daily intake of more than 12 g in women and 24 g in men), or incapacity to understand the full extent of the study.

### Evaluation of echocardiographic parameters

Echocardiographic assessment was performed according to the recommendations of the European and American societies of echocardiography.^19^ Experienced, blinded cardiologists trained in 2D and 3D echocardiography, with a focus on cardiomyopathies and heart failure, carried out a comprehensive evaluation of all cardiac parameters concentrating on left heart haemodynamic parameters within 12 h of admission and on the fifth day (after completing treatment).

Standard views were obtained from parasternal, apical, and subcostal window. Chamber dimensions were assessed using 2D method. Left ventricular ejection fraction (LVEF) and ventricular volume was calculated using the Simpson method from apical 4- and 2-chamber views. Using the pulsed and tissue Doppler method, we assessed mitral inflow and annular velocities and calculated the left ventricular diastolic function (E-Wave, E/e′ ratio and e′ velocity). Pulmonary artery systolic pressure was derived from peak tricuspid regurgitation jet velocity (continuous wave Doppler). Left atrial (LA) volume was measured in 4–and 2–chamber view (biplane) and then indexed to body surface area using the Mosteller formula [left atrial end-systolic volume index (LAESVI)]. The LA strain and stored images were analysed using software Image Arena version 4.6 (TomTec Imaging Systems GmbH, Unterschleissheim, Germany). The measurements of global strain were carried out through tracing the endocardial contour of the left atrium. The R-R gating was used as reference. In case of atrial fibrillation 5 measurements with averaging of the values were performed.^19^ Change to baseline (∆) was calculated to highlight dynamics of parameters.

### Statistical analysis

Variables are expressed as mean and 95% confidence intervals (CI). All randomized patients were analyzed and only patients without major protocol deviations took part in the per-protocol evaluation. The complete description of sample size, power, primary, and secondary analysis has been previously published.^18^ The Wilcoxon–Mann–Whitney test was performed to highlight differences [including change (∆) to baseline] between cohorts. Statistical significance was set at a level of <0.05. Statistics were performed by using SPSS version 27 (IBM SPSS Statistics, IBM Corporation, Armonk, New York, NY, USA). Graphical assessment was performed by using Microsoft Office^®^.

## Results

### Study population

Baseline characteristics of the patient cohort are summarized in *Table 1*. Median time from admission to randomization was 9.7 h. Patients were 74.7 ± 9.9 years of age and 38.2% were female. In the empagliflozin cohort, 60% of the patients showed *de novo* heart failure, while in the placebo group the proportion was 48%. Median NT-proBNP concentrations were 3386 (interquartile range 2122–5344). In the empagliflozin group, 48.1% of the patients presented with a history of atrial fibrillation, while in the placebo cohort the proportion was 51.9%.

**Table 1:** Baseline characteristics

**Parameter**	**Empagliflozin (*n* = 30)**	**Placebo (*n* = 29)**
Age in years—mean (95% CI)	72.9 (68.8–77.1)	76,5 (73.4–79.7)
Gender—*n*/*n* total (% female)	11/30 (36.7)	12/29 (41.4)
Body mass-index (kg/m^2^) mean (95% CI)	31.1 (27.5–34.7)	29.9 (27.1–32.6)
Aetiology of heart failure—*n*/*n* total (%)		
Ischaemic	8/30 (27)	10/29 (34)
Non-ischaemic	22/30 (73)	19/29 (66)
History of cardiovascular disease—*n*/*n* total (%)		
Atrial fibrillation	13/30 (43.3)	14/29 (48.2)
Diabetes mellitus type 2	13/30 (43.3)	10/29 (34.5)
Hypertension	27/30 (90)	25/29 (86.2)
Heart failure (according to LVEF) (%)		
HFrEF	13/30 (43)	14/29 (48.2)
HFmrEF	3/30 (10)	2/29 (6.9)
HFpEF	14/30 (47)	13/29 (44.9)
De novo heart failure—*n*/*n* total (%)	18/30 (60)	14/29 (48)
Valvular heart disease—*n*/*n* total (%)	15/30 (50)	17/29 (58.6)
Heart failure medication (%)		
Renin–angiotensin inhibitor	23/30 (76.7)	20/29 (69)
Sacubitril/valsartan	5/30 (16.7)	5/29 (17.2)
Mineralcorticosteroid receptor antagonist	7/30 (23.3)	4/29 (13.8)
β-Blocker	22/30 (73.3)	25/29 (86.2)
Previous treatmen with loop diuretics	19/30 (63)	17/29 (59)
Laboratory parameters—mean (95% CI)		
eGFR (mL/min)	58.2 (51–66)	62.2 (55–69)
Creatinine (mmol/L)	107 (96–118)	97.8 (87–109)
Bilirubin (µmol/L)	13.3 (9.8–16.8)	16.8 (13–20.6)
NT-proBNP (pg/mL)	4726 (2939–6513)	4823 (2923–6724)

### Primary results—baseline characteristics and atrial parameter

Echocardiographic parameters at baseline and after completing study treatment of both cohorts and the in between comparison are highlighted in *Table 2*. Mean LVEF was 43.7 ± 15.1 and 21% of the patients presented with a reduced ejection fraction under 30%. In the empagliflozin group, 50% of the patients presented with a moderate to severe valvular heart disease, while in the placebo cohort the proportion was 58.6%. Except for one patient who received empagliflozin (aortic stenosis) and one patient who received placebo (aortic regurgitation), all patients showed regurgitation of the tricuspid and mitral valve.

**Table 2:** Echocardiographic parameters at baseline and after completing study treatment

							**p**	
Parameters	**Baseline** **empagliflozin** (mean, 95% CI)	** After 5 days** **empagliflozin** (mean, 95% CI)	**Absolute Difference** **empagliflozin** (∆, 95% CI)	** Baseline** **placebo** (mean, 95% CI)	** After 5 days** **placebo** (mean, 95% CI)	**Absolute Difference** **Placebo** (∆, 95% CI)	**Baseline**	**∆**
LVEF (%)	46.9 (40.6–53.2)	46.6 (41.2–52)	0.3 (−3.4–4)	43.2 (37.8–48.6)	43.8 (38.3–49.1)	−0.6 (−2.5–1.4)	0.815	0.787
LVEDV (mL)	109 (77.9–140)	98.6 (75.6–122)	10.1 (−1.8–22)	130 (98–162)	128 (97.2–159)	2.2 (−13.8–18.2)	0.513	0.261
LVEDVI (mL/m^2)^	56.9 (41.1–72.8)	52.9 (41.1–64.7)	4 (−1.9–10.1)	64.1 (48.3–80)	62 (48–75)	2.5 (−7–12)	0.532	0.550
LVESV (mL)	59.2 (40.8–77.7)	56.7 (40.4–73)	2.5 (−7.7–12.8)	78. (54–103)	77.2 (53–101)	1.3 (−10.6–13.2)	0.352	0.547
LVESVI (mL/m^2)^	30.6 (21.2–40.1)	30.6 (21.8–39.4)	0.03 (−5.2–5.2)	37.3 (26.7–48.2)	36.5 (26.4–46.5)	0.97 (−5–7)	0.510	0.856
LVESD (mm)	38.4 (33.4–43.5)	36.5 (32.9–40.1)	1.9 (−1.1–5)	35.4 (30.5–40.3)	34.7 (30–39.4)	0.7 (−1.9–3.3)	0.329	0.679
LVEDD (mm)	52.4 (48.4–56.5)	50.2 (47–53.5)	2.19 (0.2–4.2)	51.6 (47.4–55.8)	51.4 (47.5–55.2)	0.25 (−1.7–2.2)	0.860	0.166
TAPSE (mm)	18.9 (16.4–21.5)	16.4 (14.8–18)	2.5 (−0.9–6)	16.2 (14.6–17.9)	16.4 (14.8–18)	−0.22 (−1.3–0.9)	0.083	0.077
sPAP(mmHg + CVP)	28.9 (22.8–34.9)	28.5 (19.8–37.3)	0.4 (−6.9–7.6)	31.9 (28–35.9)	28.1 (22.7–33.6)	3.9 (−0.7–8.5)	0.269	0.335
E (cm/s)	94.1 (82.8–105)	94.2 (82.6–106)	−0.1 (−11.3–11.2)	109 (94.2–125)	104 (88–121)	5.5 (−8.8–19.8)	0.140	0.229
E/e´ ratio	16.7 (14.8–18.5)	15.2 (13.6–16.9)	1.4 (−0.8–3.7)	16.6 (13.4–19.8)	17.3 (14–20.6)	−0.7 (−2.8–1.4)	0.408	0.088
LA Strain (%)	4.9 (3.2–6.6)	6.6 (3.6–9.6)	−1.7 (−4.1–0.7)	5.7 (2.4–9)	6.1 (3.7–8.5)	−0.4 (−3.1–2.3)	0.589	0.361
LAV (mL)	109 (95.9–122)	78 (67.7–88.3)	30.9 (20.1–41.7)	113 (91.6–134)	103 (82.5–123)	10.5 (0.4–20.5)	0.975	**<0.001**
LAESVI (mL/m²)	55.5 (48.1–62.9)	39.8 (34.1–45.5)	15.7 (9.8–21.6)	55.7 (44.7–66.8)	50.6 (40.3–60.8)	9.7 (5.7–13.6)	0.381	**0.016**

*P*-values represent inter-cohort statistical evaluation (baseline represents the statistical evaluation at baseline and ∆ represents the statistical evaluation of the difference after 5 days of treatment to baseline). Values are shown as mean and 95% CI. (LVEF, left ventricular ejection fraction; LVEDV, left ventricular end-diastolic volume; LVEDVI, left ventricular end-diastolic volume index; LVESV, left ventricular end-systolic volume; LVESVI, left ventricular end-systolic volume index; LVESD, left ventricular end-systolic diameter; LVEDD, left ventricular end-diastolic diameter; TAPSE, tricuspid annular plane systolic excursion; sPAP, systolic pulmonary artery pressure; CVP, central venous pressure; LA Strain, left atrial strain; LA-Volume, left atrial volume; and LAESVI, left atrial end-systolic volume index.

**Figure 2:**
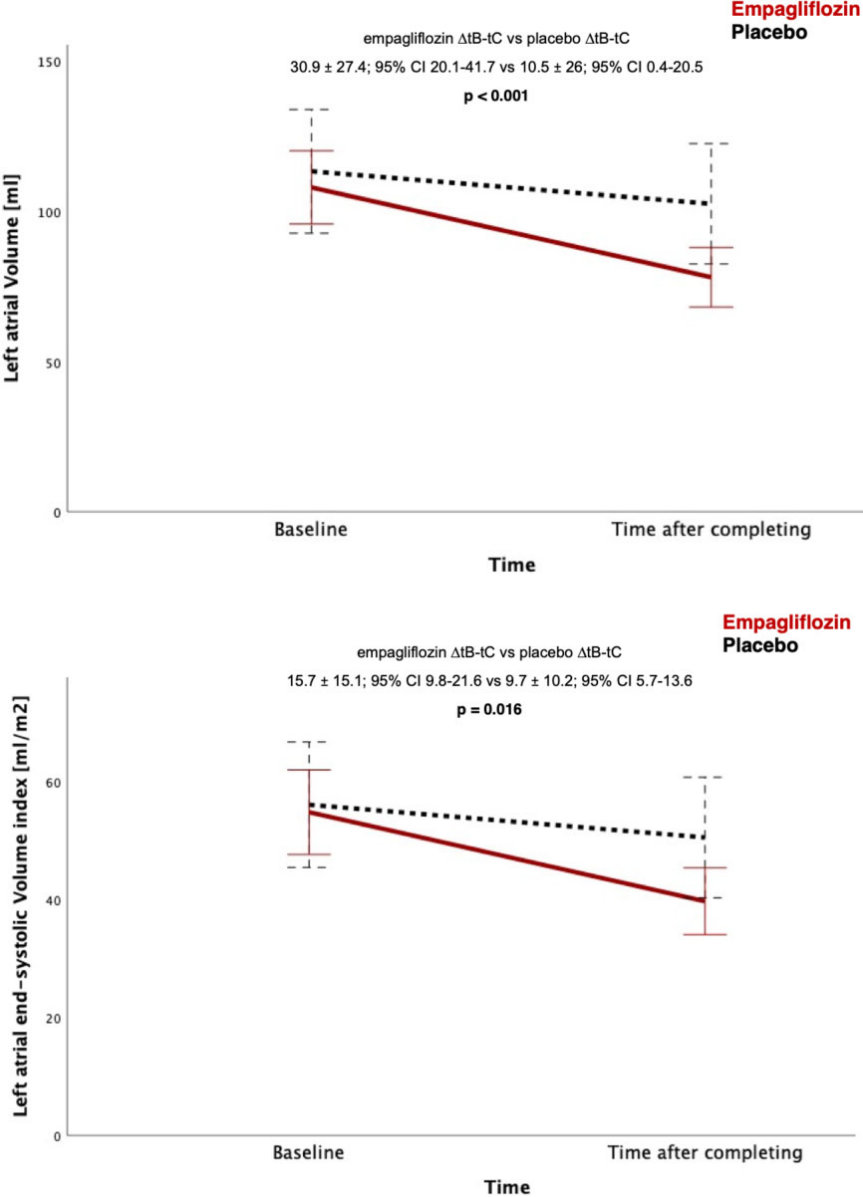
Trends in LAV (top) and LA end-systolic volume index (bottom). Data are highlighted as mean and bars represent 95% CI (time after completing = after 5 days).

At baseline, there was no difference between groups in all assessed parameters. After decongestion, we could demonstrate a decrease in left atrial volume (LAV) (mL/m^2^) in both cohorts. The change after completing study treatment compared to baseline evaluation (∆tB-tC) presented a statistically significant reduction (*P* = <0.001) in the empagliflozin cohort (∆tB-tC = 30.9 ± 27.4; 95% CI 20.1–41.7) compared to placebo (∆tB-tC = 10.5 ± 26; 95% CI 0.4–20.5). The reduction in LAESVI presented a statistically significant result (*P* = 0.016) as well (empagliflozin ∆tB-tC = 15.7 ± 15.2; 95% CI 9.8–21.6 vs. placebo ∆tB-tC = 9.7 ± 10.2; 95% CI 5.7–13.6). This aspect is highlighted in *Figure 2*. Left atrial strain showed no statistically significant changes after 5 days of treatment (*P* = 0.361).

### Secondary results—ventricular parameter

The E/e′ ratio showed a reduction in the empagliflozin cohort, whilst the placebo cohort presented an increase after treatment. All other parameters presented no statistically significant changes after completing study treatment (*Table 2*), including LVEF (*P* = 0.787), tricuspid annular plane systolic excursion (TAPSE) (*P* = 0.077), systolic pulmonary artery pressure (sPAP) (*P* = 0.335), and E-Wave (*P* = 0.229).

As formerly published,^18^ empagliflozin increased diuresis in patients with ADHF irrespective of LVEF or the presence of diabetes by 28.9% compared to placebo.

## Discussion

This study shows for the first time in a randomized setting that SGLT2 inhibition through empagliflozin achieves a relevant decrease in LAV and LAESVI in patients undergoing recompensation for acute heart failure. These echocardiographic findings support data on pharmacologic unloading through increased diuresis from the EMPAG-HF-Study,^18^ which detected an increase in urine output through addition of empagliflozin during acute recompensation without renal injury. It is the first randomized study to highlight primary benefits of additive treatment with empagliflozin in patients with ADHF. This pre-defined subanalysis highlights the advantage of empagliflozin in reducing LAV and LAESVI as additive treatment versus placebo. From a mechanistic point of view, SGL2 inhibitors achieve decongestion through osmotic diuresis and natriuretic effects, intravascular volume depletion, and positive glomerular feedback.^20^ Clinical trials have demonstrated a possible impact on clinical outcome, especially regarding decongestion and preventing rehospitalization.^21,22^ Recently, a multicentre observational registry showed an improvement of cardiovascular outcome and myocardial remodeling in type 2 diabetes mellitus patients with severe aortic stenosis and an LVEF <50% who received SGLT2 inhibitors compared to patients who received other antidiabetic therapy. It could be demonstrated that SGLT2 inhibition had a greater impact on LVEF recovery (especially in patients with a baseline LVEF ≤30%), reduction of LVEDV, and sPAP after transcatheter aortic valve implantation.^23^

Many previous studies have highlighted the prognostic value of LAESVI^24–28^ irrespective of the underlying pathology. The LA represents a dynamic structure, which can highlight not only the filling status of a patient but also indicate specific haemodynamic aspects such as ventricular filling. The American Society of Echocardiography and the European Association of Cardiovascular Imaging recommend LAV assessment,^19^ as a pivotal predictor of mortality and rehospitalization in heart failure. Our imaging data suggest that empagliflozin achieves faster restoration of euvolaemia in acute heart failure patients independently of the left ventricular function or the history of heart failure. To our best knowledge, no other trial has shown this important aspect in ADHF.

Another important aspect is the impact on left ventricular unloading. The trend for lower E/e′ reflects it as a parameter of decongestion. While mechanical unloading plays an important role with increasingly evidence,^29^ medical therapy needs novel and effective strategies. In the past, captopril^30^ has been shown to reduce left ventricular pressure in ischaemic heart injury, but data of decongestive therapy and its unloading effectiveness are inconsistent. As previously shown, dilatation of left atrium represents a strong correlation to left ventricular pressure and pulmonary capillary wedge pressure.^31^ Through its decongestive effects, empagliflozin may reduce left ventricular pressure and achieve unloading in acute heart failure.

Furthermore, it could lead to a reduction of atrial fibrillation burden and rhythm stability.^32–34^ As previously shown, the presence and development of atrial fibrillation in HF leads to a worsening prognosis.^35^ The increase in mortality is independent of LVEF.^36^ Our data implicate a faster reduction in LA pressure under empagliflozin along with enhanced diuresis. This result is supported by the primary results of the EMPAG-HF trial, which presented a statistically significant decrease in NT-proBNP.^18^ However, the impact on atrial fibrillation burden needs further analysis on a larger cohort with longer follow up and appropriate statistical power.

Several aspects differ in this secondary analysis from prior trials on SGLT2 inhibitors in heart failure. First, no prior trial has evaluated echocardiographic changes in ADHF patients and additive treatment with empagliflozin. Second, imaging data reflect benefits and safety profile of SGLT2 inhibition regarding the acute phase of cardiorenal recompensation.^18^ Third, stability of other aspects such as chamber quantification, diastolic, and valvular function was monitored at baseline and after cessation of study medication, at time points that comprise the clinically vulnerable phase of acute heart failure.

Finally, we should highlight differences with the EMPULSE trial, one of the latest and most important trials in ADHF. EMPAG-HF trial randomized patients within 12 h following hospital admission, while in the EMPULSE trial, patients were randomized between 12 h and 5 days (mean: 3 days). Another aspect is the choice of 25 mg empagliflozin per day in EMPAG-HF (vs. 10 mg per day in EMPULSE). The higher dosage in the EMPAG-HF trial was chosen to evaluate not only the impact in urine output but also the safety of such a therapeutic regime. The primary outcome of the EMPULSE trial was the clinical benefit at 90 days, while the EMPAG-HF assessed total cumulative urine output over 5 days. Both studies demonstrated the efficacy in clinical aspects and safety especially regarding renal parameters. The pre-defined secondary analysis of the current manuscript evaluated the impact of additive empagliflozin treatment (25 mg/day) in echocardiographic parameter.

### Study limitations

Our data are derived from a prospective, randomized double-blind, placebo-controlled single-centre study and need further exploration in larger cohorts with a longer treatment, follow-up time, and adequate statistical power. This represents the main limitation of our study. Furthermore, our findings need confirmation through multimodal imaging such as cardiac magnetic resonance imaging or invasive haemodynamics, to evaluate the correlation with non-invasive imaging. Additionally, these results need evaluation also in the cardiac shock patients (hypoperfusion syndrome) cohort, to evaluate the role of empagliflozin in combination with inotropes regarding decongestion, reduction of pulmonary oedema, and renal function stability. In this context, the role and prognostic value of SGLT2 Inhibitors is still unknown. Last, we should highlight that the enrollment phase was marked and affected by the COVID-19 pandemic and the results of our study might have been determined by related external aspects.

## Conclusions

The addition of 25 mg/day empagliflozin to standard therapy reduces LAV and LAESVI in ADHF patients. These aspects correlate with the increase in urine output after 5 days of treatment without renal injury, as reported from the EMPAG-HF trial.

## Clinical perspectives

The significant enhancement in urine output under additive treatment with empagliflozin finds confirmation in our imaging data and translate not only in physiological but also in haemodynamic and morphologic effects.

While the EMPAG-HF trial showed the efficacy and safety vs. placebo, we could demonstrate the acute reduction in LAV and LAESVI as a morphologic and haemodynamic correlate.

This could add to the prognostic beneficial effects of empagliflozin regarding early initiation and acute dynamic changes in left heart filling parameters. These aspects can represent a future approach in ADHF patients.

## What is new?

This pre-defined secondary analysis presents for the first time statistically significant reductions in LAV and LA end-systolic volume index after early initiation of empagliflozin in ADHF patients.

No new therapeutic strategies have shown changes in LA and left ventricular filling parameters in a randomized setting.

## Notes

**Conflict of interest**: P.C.S. received honoraria and travel support from Bayer, AstraZeneca, Daiichi Sankyo, Novartis, Actelion, Roche, Sanofi Aventis, Pharmacosmos, Medtronic, Thoratec, Boehringer Ingelheim, HeartWare, Coronus, Abbott, Edwards Inc, Boston Scientific, St Jude Medical, Abiomed, and the German Cardiac Society; research support from the National Institutes of Health, the German Research Foundation, the Else Kröner Fresenius Foundation, the German Heart Foundation, the European Society of Cardiology, Actelion, Medtronic, BMBF, Abiomed, Boehringer Ingelheim, and Boston Scientific; and served on advisory boards for the German Research Council, Eurotransplant, Novartis, Bayer, Pharmacosmos, AstraZeneca, Boehringer Ingelheim Inc, the German Cardiac Society, and the European Society of Cardiology.

S.M.-W. received honoraria and travel support from Bayer, Daiichi Sankyo, Novartis, Boston Scientific, Abiomed, and the German Society of Cardiology; and research support from Boston Scientific and Abiomed.

Dr von Haehling has been a paid consultant for or received honoraria payments from AstraZeneca, Bayer, Boehringer Ingelheim, BRAHMS, Chugai, Grünenthal, Helsinn, Hexal, Novartis, Pharmacosmos, Respicardia, Roche, Servier, Sorin, and Vifor; and reports research support from Amgen, AstraZeneca, Boehringer Ingelheim, IMI, and the German Center for Cardiovascular Research (DZHK).

M.B. has been a paid consultant for or received honoraria payments or travel support from Novartis, AstraZeneca, Boehringer Ingelheim, Vifor Pharma, OTSUKA, Bristol Myers Squibb, and Pfizer.

The other authors report no conflicts.

## Data availability

The data underlying this article will be shared on reasonable request to the corresponding author.
